# UniProt-DAAC: domain architecture alignment and classification, a new method for automatic functional annotation in UniProtKB

**DOI:** 10.1093/bioinformatics/btw114

**Published:** 2016-03-07

**Authors:** Tunca Doğan, Alistair MacDougall, Rabie Saidi, Diego Poggioli, Alex Bateman, Claire O’Donovan, Maria J. Martin

**Affiliations:** European Molecular Biology Laboratory, European Bioinformatics Institute (EMBL-EBI), Hinxton, Cambridge, UK

## Abstract

**Motivation:** Similarity-based methods have been widely used in order to infer the properties of genes and gene products containing little or no experimental annotation. New approaches that overcome the limitations of methods that rely solely upon sequence similarity are attracting increased attention. One of these novel approaches is to use the organization of the structural domains in proteins.

**Results:** We propose a method for the automatic annotation of protein sequences in the UniProt Knowledgebase (UniProtKB) by comparing their domain architectures, classifying proteins based on the similarities and propagating functional annotation. The performance of this method was measured through a cross-validation analysis using the Gene Ontology (GO) annotation of a sub-set of UniProtKB/Swiss-Prot. The results demonstrate the effectiveness of this approach in detecting functional similarity with an average F-score: 0.85. We applied the method on nearly 55.3 million uncharacterized proteins in UniProtKB/TrEMBL resulted in 44 818 178 GO term predictions for 12 172 114 proteins. 22% of these predictions were for 2 812 016 previously non-annotated protein entries indicating the significance of the value added by this approach.

**Availability and implementation:** The results of the method are available at: ftp://ftp.ebi.ac.uk/pub/contrib/martin/DAAC/.

**Contact:** tdogan@ebi.ac.uk

**Supplementary information:** Supplementary data are available at *Bioinformatics* online.

## 1 Introduction

The reduction in the cost of sequencing has led to the accumulation of a vast amount of data in biological databases. These data are stored in public repositories such as the UniProt Knowledgebase (UniProt Consortium, 2015) for protein sequences, and NCBI GenBank (Benson *et al.*, 2008) and the EMBL Nucleotide Archive (Leinonen *et al.*, 2011) for gene sequences. In order to make sense of these data, the stored sequences need to be annotated with respect to their functional and evolutionary properties. Defining the functions of genes and gene products is a difficult task due to the biological complexity of organisms. There are various projects aiming to standardize the description of the functional attributes of biological sequences by introducing controlled vocabularies. The Gene Ontology (GO) project provides the most comprehensive functional standardization system for proteins (Gene Ontology Consortium, 2015). GO uses a directed acyclic graph (DAG) structure to define the functions from generic to specific in three main categories namely: molecular function, biological process and cellular component.

Discovery of functional properties for proteins is a key step in biomedical research, yet experimental identification of proteins is still a quite laborious and expensive task. This has led to many similarity-based computational methods being developed to infer the unknown properties of proteins based on their similarity to experimentally annotated proteins. The most widely used approach is sequence alignment (Altschul *et al.*, 1990; Pearson and Lipman, 1988). A significant proportion of the unknown functional space has been covered thanks to this procedure where similarity is inferred in terms of the shared evolutionary history of the sequences.

Nevertheless, different approaches have been tried lately for the prediction of protein properties, to augment the performance of sequence methods. One approach is to exploit information on the physicochemical properties of the amino acids in the protein sequence to infer subcellular localization (Sarda *et al.*, 2005). A different approach is the prediction of the structure of the proteins using their sequences (Drozdetskiy *et al.*, 2015; Söding *et al.*, 2005). Yet another one is to identify evolutionary conserved regions in the sequences (such as motifs and domains) and to relate these sequence segments to specific functions (Bailey *et al.*, 2009; Doğan and Karaçalı, 2013; Tompa *et al.*, 2014). The idea is that genes with mutations in these regions are selected against because changes in these functionally active segments may cause a decrease in efficiency or even the loss of function, decreasing the fitness of the gene. Combinatorial approaches are gaining popularity, where different methods are combined to increase the coverage and the quality of predictions; either by gathering together different features under one classifier (Chou, 2011) or by using multiple classifiers at the same time (Saraç *et al.*, 2010). Critical Assessment of Protein Function Annotation was initiated in 2011, in order to evaluate various methods in terms of their performance in the prediction of GO terms on a standard dataset (Radivojac *et al.*, 2013).

Many of the methods using evolutionary conserved sub-sequences focus on protein domains. These are the structural building blocks in proteins that are able to function and fold independently from the rest of the protein (Wetlaufer, 1973). There are many well-established biological databases dedicated to the identification and search of functional domains and the grouping of similar protein sequences into families. These databases attempt to assign functional annotations to the domains and families, and approach a protein sequence as a functional combination of its domains. Some of the widely used sequence-based domain/family databases are Pfam (Finn *et al.*, 2014), PROSITE (Sigrist *et al.*, 2012), HAMAP (Pedruzzi *et al.*, 2015), SUPERFAMILY (Wilson *et al.*, 2009) and InterPro (Mitchell *et al.*, 2014). InterPro incorporates all of the above databases and more to provide a comprehensive classification of proteins.

One view in the field of protein function inference states that the function of a protein is not simply the sum of the functions of the independent domains it contains, but rather is a unique property emerging from the contribution of all of the structural blocks synergistically (Bashton and Chothia, 2007). This has led to the concept of domain architectures/arrangements (DA) defined as the organizational properties of a protein regarding the domains it contains. These properties may include the domain content, linear order of the domains in the protein sequence and recurrence of the domains in the protein. In DA-based methods, statistically significant similarities between test proteins are identified using the above-mentioned properties.

Examples of DA information being employed in biological data analysis methods include Björklund et al. (2005), Geer et al. (2002), Lin et al. (2006) and Song *et al.* (2007). Although the methodology used varies greatly between the different studies, most of them try to predict the pairwise similarities/homologies between proteins (Lin *et al.*, 2006 and Song, *et al.*, 2007). Earlier studies mostly focused on similarities in the domain content of proteins (Geer *et al.*, 2002), whereas information regarding domain order, position, recurrence and promiscuity is more frequently used in later studies (Fang and Gough, 2013; Kummerfeld and Teichmann, 2009; Lee and Lee, 2009; Messih *et al.*, 2012; Song *et al.*, 2007; Terrapon *et al.*, 2014). The study by Björklund et al. (2005) was the first to incorporate information about the sequential order of domains into the similarity search (Björklund *et al.*, 2005). In most of these studies, the authors set out to quantify the similarities between proteins using domain information, mostly with the aim of identifying pairwise homologies.

Here, we present the UniProt Domain Architecture Alignment and Classification (DAAC) procedure for the automatic annotation of uncharacterized proteins in UniProtKB based upon domain architectural similarity to manually reviewed sequences in the UniProtKB/Swiss-Prot. Four attributes are incorporated into the measurement of domain architectural similarity: domain content, order, position and recurrence. The proposed method incorporates domain annotation from InterPro in order to obtain comprehensive domain information coverage for the proteins.

This study is the first that we are aware of to use DA comparison and classification in the automatic functional annotation of large protein sets. The proposed method also brings new approaches to the field by (i) employing InterPro as the domain annotation source, (ii) the use of multi-label classification technique to annotate proteins with multiple functions in one run and to be able to optimize the parameters for each functional term independently and (iii) application of domain weights during the alignment step to direct the procedure to the optimal solution. Multi-label classification is a technique used to classify each sample into one or more classes. This method is frequently employed to analyze examples with multiple attributes as opposed to the traditional single-label or binary classification (Tsoumakas *et al.*, 2010). In the latter, each of the objects under consideration can be assigned to only one class among a set of mutually exclusive classes. However, in the real world, objects can belong to multiple classes that are not necessarily disjoint, e.g. a protein may interact with ATP (attribute 1) in the plasma membrane (attribute 2). There are two ways to address these cases. The first is to perform several separate processes of binary classification, each for one class, and then to aggregate the results. The second option is to perform a multi-label classification, which allows assigning an object to more than one class and in one single process.

The proposed method has been validated using protein sequences from UniProtKB/Swiss-Prot together with their experimentally validated GO annotations. By doing so, we hope to demonstrate that the results of the analysis have biological relevance for protein function prediction. Finally, we applied the method to UniProtKB/TrEMBL to obtain functional predictions for the protein entries in the database.

It is important to emphasize that the proposed method is not designed to replace conventional sequence-based methods but to complement them. The case for using DA comparison methods to complement sequence-based approaches has been reported in previous studies (Lee and Lee, 2009; Messih *et al.*, 2012; Terrapon *et al.*, 2014).

## 2 Methods

**Fig. 1.**
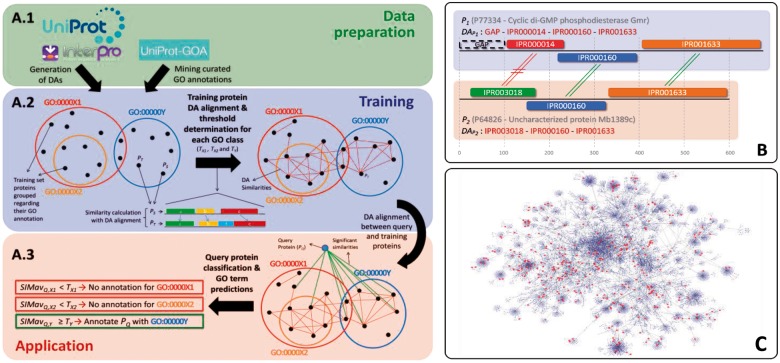
(**A**) Schematic representation of the method; (**B**) Representation of pairwise DA alignment between two proteins; (**C**) GO MF DAG; nodes: all terms (blue), predicted terms (red)

Figure 1A displays the representation of DAAC. The method first generates the DAs for both training and test proteins as explained in Section 2.1. Training of the system takes place as the second step where DAs of reference proteins are aligned pairwise in an all-against-all manner using the InterPro domain hits as the strings instead of amino acids. Then training proteins are grouped under classes representing unique GO terms. Class-specific similarity thresholds are determined at the end of the training (as discussed in Section 2.3). The third step is the application. DAs of the query proteins are aligned to the training samples and classified into the annotation term classes considering their similarity measure from the alignment. Query proteins receive the GO term of the corresponding classes as predictions.

### 2.1 Generation of the DAs

DAs are generated using the InterProScan results for UniProtKB proteins. InterPro combines information on protein attributes from 11 different Consortium member databases. The individual components of this information are sequence signatures responsible for certain properties of the protein. Signatures from the different member databases are merged under distinct entries in InterPro by manual curation (Mitchell *et al.*, 2014).

In this way more than 35 000 signatures are integrated into InterPro(v.49) from the different member databases; resulting in 7518 domain, 18 218 family, 277 repeat and 847 site type InterPro entries.

In order to generate the DAs, v.49 release files were downloaded from the InterPro database. DAs were generated using the InterPro domain type hits to UniProtKB proteins. A DA is composed of the linear arrangement of domains in the protein sequence from the N to C terminus. In addition, non-annotated regions in the sequences longer than 30 amino acids are annotated with hypothetical ‘GAP’ domains in DAs. The reason behind is that these regions may contain domains that are yet to be identified. Two sample proteins from UniProtKB (P77334 and P64826) and their DAs are shown in Figure 1B.

### 2.2 Weighted DA alignment

DA alignment is the pairwise optimal alignment between two proteins using the linear arrangement of their domains instead of amino acids. The Needleman-Wunsch Global Sequence Alignment algorithm (Needleman and Wunsch, 1970) is the core of the proposed DA alignment algorithm. However, the algorithm has been modified to carry out global alignment using DAs. The algorithm employs more than 7500 distinct InterPro domains as its alphabet as opposed to 20 kinds of amino acids in the conventional sequence alignment. Alignments are scored using equal values (with opposite signs) for matches and mismatches, half of the mismatch value for gap openings and half of the gap opening value for gap extensions.

Domain hits are weighted before the alignment procedure in order to reduce the contribution of promiscuous domains (the ones appearing in a variety of proteins families and have minimal effect on the total function of the protein) to the final similarity measure. Inverse domain frequency is a measure of how frequently a domain appears in different proteins. Highly frequent domains appearing in various non-related proteins are less informative compared with the rarely occurring domains (Song *et al.*, 2007). As a result, frequent domains are weighted less in order to decrease their contribution to the scoring. Inverse domain frequency is defined as:

(1) $$A_{d}={\log\;}_{2}\frac{N_{t}}{N_{d}}$$

where *N_t_* is the total number of proteins in the set and *N_d_* is the number of proteins containing domain *d*. To reduce the computational burden, scoring matrices are generated on the fly during the alignment, only using the domains in the test protein pair. The substitution values for each domain pair in the raw scoring matrix are multiplied by the inverse domain frequency values of both domains in the corresponding pair prior to the alignment to obtain the final scoring matrix. An example pairwise DA alignment is shown in Figure 1B. Alignment score is calculated considering gaps, matches and mismatches. The following equation displays the score calculation for the example in Figure 1B:

(2) $$S_{1,2}=Gop_{p_{2}}+I_{IPR\;000014,\;IPR\;003018}+M_{IPR\;000160}+M_{IPR001633}$$

where *Gop_2_* represents gap opening penalty for the second protein, *I* is the mismatch score between the corresponding InterPro domains and *M* is the match score for the corresponding InterPro domain. Any matches between an actual domain and a GAP domain or between two GAP domains are mildly negatively scored with gap opening penalties, instead of mismatches, since there is no knowledge about the information hidden in these regions. The finalized DA similarity (between 0 and 1) is obtained by normalizing the alignment score using the self-alignment scores of the two DAs. Thus, alignment scores from various DA pairs become comparable with each other. The DA similarity between two protein 1 and 2 is:

(3) $$SIM_{1,2}=\min\;\left[\frac{S_{1,2}-S_{mn_{1,2}}}{S_{self_{1}}-S_{mn_{1,2}}},\frac{S_{1,2}-S_{mn_{1,2}}}{S_{self_{2}}-S_{mn_{1,2}}}\right]$$

where *S_1,2_* is the DA alignment score, *Smn_1,2_* is the minimum alignment score that could be obtained from these two proteins and *Sself_1_* is the self-alignment score of protein 1. The minimum alignment score is calculated as if the two proteins have no common domains. This is done by the placement of gaps in sequence 1 equal in number to the number of domains in sequence 2, followed by the placement of gaps in sequence 2 equal in number to the number of domains in sequence 1; and calculating the total negative score for this alignment. Placing gaps is favored over mismatches here due to the values of the selected penalties. The reason for incorporating the minimum alignment score in the equation is to compensate for negative alignment scores that would otherwise result in negative DA similarity values.

### 2.3 Classification and function prediction

The reference data for GO term prediction was composed of the DAs for the protein entries in the UniProtKB/Swiss-Prot (v2014_11) and the associated GO annotation (with experimental evidence codes) taken from the UniProt-GOA database (Dimmer *et al.*, 2012). Evidence codes marked as ‘experimental’ in the GO system (codes: EXP, IDA, IPI, IMP, IGI and IEP) are of the highest quality and reliability. After mining the dataset from UniProt-GOA, the annotations are extended to include all parents of the terms found, excluding the root (top level) terms for all GO categories.

For the training/learning step, the DAs of proteins bearing experimentally validated GO annotation are grouped into unique GO term classes. Here, each class represents a specific GO term and the proteins containing the corresponding annotation are the members of the class (Fig. 1A.2). The DAs of all reference proteins are aligned to each other in an all-against-all manner and their similarities are calculated using the procedure explained in Section 2.2. Next, class-specific similarity thresholds are determined. For each GO class we run the cross-validation process using similarity thresholds varying from 0 to 1 with 0.02 increments. An F-score value is calculated for each threshold and the one yielding the best performance is selected as the similarity threshold for the corresponding GO class. Modeling each GO term as an independent classifier provides the means to optimize their thresholds. In the end, specific GO terms usually have high threshold values; whereas, generic GO terms tend to obtain low values. These thresholds are later used for the classification decision during the application phase.

**Fig. 2.**
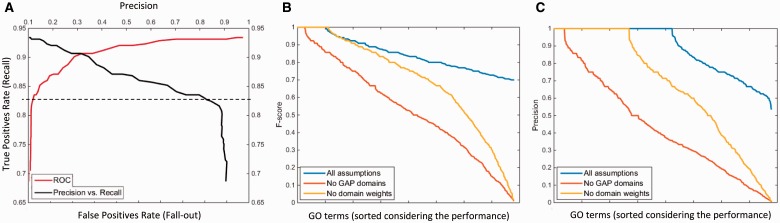
Cross-validation results: (**A**) ROC and precision versus recall curves for a GO term class; (**B**) Performance of the method as F-score and (**C**) as Precision (Color version of this figure is available at *Bioinformatics* online.)

Considering the application step: classification of proteins is carried out with the help of the reference data and the pairwise DA alignment similarity values. Following the alignment of a query protein’s DA to all members of a class (Fig. 1A.3), the mean DA similarity (*SIMav_Q,Y_*: for protein *Q* to class *Y* in the example) of the test protein to the GO term class is calculated. For the cases where this similarity exceeds the class-specific similarity threshold (*T_Y_*), the method classifies the test protein into the corresponding class and the term is given as a prediction for the query protein. The procedure is carried out using all training classifiers, with a multi-label classification approach. A query protein can be classified to more than one class and thus has multiple labels.

**Fig. 3.**
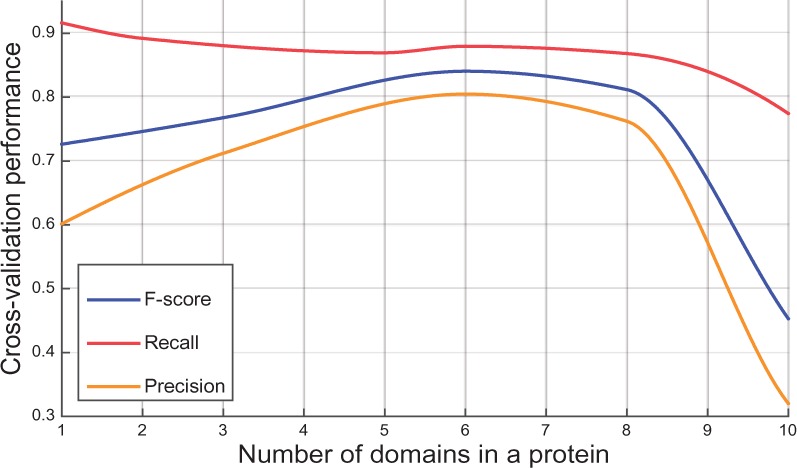
Number of domains per protein versus performance in cross-validation graph (Color version of this figure is available at *Bioinformatics* online.)

## 3 Results and discussion

Currently DAs are generated for all UniProtKB records at each release and stored in the UniProt Domain Architecture Database. Table 1 shows the statistics for the DA generation process separately for the UniProtKB/Swiss-Prot and UniProtKB/TrEMBL databases (v2014_11). As observed from Table 1, 74% and 64% of UniProtKB/Swiss-Prot and UniProtKB/TrEMBL entries, respectively, are covered with DAs. Also from Table 1, the number of unique DAs generated in UniProtKB/Swiss-Prot is ∼13% of the number of entries with domain hits. This rate is only 2% for UniProtKB/TrEMBL, and the reason for this can be attributed to the higher redundancy in UniProtKB/TrEMBL compared to UniProtKB/Swiss-Prot.

**Table 1.** Statistics of the DA generation on UniProtKB databases

Database UniProtKB/:	Swiss-Prot (v2014_11)	TrEMBL (v2015_12)
No. of input protein entries:	547 084	55 270 679
No. of entries with InterPro domain hits:	407 247	35 564 711
No. of unique DAs generated:	54 388	1 148 372

### 3.1 Performance of the method

A cross-validation experiment was carried out in order to observe the performance of DAAC on data with known labels (methodological details of this run are given in the Supplementary Information). Figure 2A shows the ROC and precision versus recall curves for an example GO term class: Endopeptidase activity (GO:0004175) and the performance at the selected class-specific optimum similarity threshold (marked with the dashed line). It should be noted here that it would not be possible to display the overall performance of the method in an ROC curve because each term was evaluated separately as an independent classifier. Figure 2B and C display the performance of the method in the cross-validation procedure where each value on the horizontal axis represents a different GO term and the vertical axis corresponds to the performance measure for these terms in F-score and precision, respectively (GO terms are sorted in descending order according to performance).

The performance of the method was calculated using the statistical measures explained in the Supplementary Information. The method performed well on 778 GO terms (F-score > 0.7) out of 13 826 tested terms with a mean recall: 0.84, precision: 0.89 and F-score: 0.85, and the high-performance GO term set was composed of 536 molecular function, 82 cellular component and 160 biological process terms. Only the predictions for these 778 terms are considered during application on UniProtKB/TrEMBL. In Figure 2B and C, the performance of the overall method for the high performance GO classes is shown by the black curve (blue curve in online version). Precision is displayed in Figure 2C to show the low number of false positives for the selected GO classes; 675 of the 778 classes have a precision >0.7. Additionally, the area under the ROC curve (AUC) has been calculated. Because each GO term class is independent and has its own ROC curve, we have calculated an AUC value for each one, giving an overall mean value of 0.88  ±  0.10. The mean AUC for non-selected (low performance) GO term classes was calculated as 0.68 ± 0.15.

In order to observe the effect of adding imaginary GAP domains in DAs and the effect of weighting the domains on the performance, the cross-validation experiment was repeated without making these adjustments. The dark and light gray curves (red and yellow curves in online version) in Figure 2B and C correspond to the performance without the GAP domains and without the domain weighting, respectively. Omitting GAP domains reduces the performance significantly in terms of both F-score and precision (recall curves are given in Supplementary Fig. S2). The mean AUC for this run was 0.8 ± 0.10 (9% reduction compared to the normal procedure) and only 293 GO terms were marked as high performance classes. Omitting the domain weights resulted in a slight reduction in performance with 706 GO terms marked as high performance classes and a mean AUC of 0.87 ± 0.10 (1% reduction compared to the normal procedure). The results indicate that both including imaginary GAP domains in DAs and weighting domains had a positive effect on the performance of the method in cross-validation. Performance comparison within different GO categories is discussed in the Supplementary Information.

Figure 1C shows the entire GO DAG for molecular function category. Blue and red nodes represents all GO terms and the ones predicted by DAAC with high performance respectively; and the gray edges correspond to the direct relations between the terms. As observed from Figure 1C the terms predicted by DAAC are distributed among the whole graph. This indicates that the DAAC approach is global considering the function space and the method has potential to predict the functions of proteins from various families.

### 3.2 Performance versus protein complexity

Up to this point the performance of the method has been measured in terms of classifiers (GO terms). Another important topic here is testing the system in terms of input samples (proteins). Exploration of DAAC performance on proteins with varying complexity is one way to observe if the method fails on test samples with certain attributes. The number of domains on a protein can be employed to measure the complexity. In order to observe how the method performs with changing number of domains on proteins, we divided the cross-validation results by the number of domains contained in each protein. Figure 3 displays the F-score, recall and precision values from this analysis. As observed, recall is at its maximum with single-domain proteins, is generally stable up to nine domains and starts to decrease afterwards; F-score and precision have similar trends, rising with increasing number of domains, peaking around 5–7 domains and starting to decrease after this point. The overall performance peak with six domains is attributed to the method being based on architectural similarities between multi-domain proteins. Normalized DA similarity values between single and multi-domain proteins usually remain higher compared with the alignment of two multi-domain proteins if the only domain in the single-domain protein is matched. This sometimes results in excessive propagation of annotations and thus, results in an elevated recall but a lowered precision for single domain proteins. Figure 3 shows that the performance is acceptable for the single domain proteins (F-score: 0.73), however, similarity detection over complex architectures provides better performance (F-score: 0.85). The decrease after eight domains per protein can be explained as the functions of these proteins become extremely complex so that the method fails to capture the underlying signature combination. Therefore, predictions for proteins with nine domains or higher were considered un-reliable and input samples with this attribute were removed from the query set in the data preparation step.

### 3.3 UniProtKB/TrEMBL annotation

The method is run on UniProtKB/TrEMBL to annotate nearly 55.3 million uncharacterized protein entries in this database. The statistics of this run are shown in Table 2 as the number and percentage of predictions and proteins (in brackets). The total of percentages (for proteins) exceeds 100 because proteins may have multiple predictions. The output predictions are compared with the current GO term annotations from automatic annotation systems in UniProtKB/TrEMBL to observe the correspondence of DAAC with other systems and to see if there is an added value in this approach. In Table 2, ‘new’ predictions means the predictions given on previously non-annotated proteins; ‘identical’ refers to the predictions that are the same as the ones in the current database; ‘similar’ predictions are those having a parent–child relationship with the GO term annotation for that particular protein in the database; and lastly ‘differential’ predictions are the ones that are unrelated to the current annotations of that protein in the database.

**Table 2.** Statistics of DAAC application results on UniProtKB/TrEMBL and comparison to the current annotation in the database

	Predictions (No. of proteins in brackets)	Ratio (on % of proteins)
Total no. of:	44 818 178 (12 172 114)	100% (100%)
No. of new:	10 020 251 (2 812 016)	22% (23%)
No. of identical:	6 607 303 (5 065 640)	15% (42%)
No. of similar (total):	20 755 459 (7 342 619)	46% (60%)
No. of similar (specific):	15 358 089 (5 877 438)	34% (48%)
No. of similar (generic):	4 966 612 (2 879 775)	12% (24%)
No. of differential:	7 435 165 (3 303 747)	17% (27%)

Coverage increase in UniProtKB/TrEMBL database: 8.0%.

As shown in Table 2, 2 812 016 of the previously non-annotated protein entries received a GO term prediction from DAAC. These results indicate the value of the DA-based approach. The low percentage of identical predictions can be attributed to the fact that most of the current annotations in the database are given for generic GO terms; however, DAAC mostly predicts very specific terms. This also explains the relatively high ratio of similar predictions (46%). In 74% of the similar prediction cases DAAC predicted a more specific GO term compared to the database annotation. The differential prediction ratio of 17% can be considered acceptable for a comparison between different automated prediction systems; however, there also is an ongoing work to survey the differential predictions between various predictions systems in UniProt. It is important to note that only the classifiers (GO terms) with a high validation performance (F-score > 0.7) were included in these runs. In this way we tried to avoid giving false positive predictions as much as possible.

### 3.4 Biological inspection of the results

In order to comment on the biological relevance of the results and the value added by the method, we would like to discuss two interesting example cases here. The first example case: GO:0004653 (polypeptide N-acetylgalactosaminyltransferase activity) is a molecular function category term that is associated with two very similar DAs by the DAAC method as shown in Table 3. The domains IPR001173 and IPR000772 appear together in both of these architectures. Taken individually, the InterPro entries have broad specificity. IPR001173 (‘Glycosyltransferase 2-like’) is found in a diverse family of glycosyl transferases. IPR000772 (‘Ricin B lectin domain’) identifies a galactose binding property found in a wide range of enzymes and recognition proteins. However the combination of the two domains is a particular feature of the UDP-GalNAc:polypeptide α-N-acetylgalactosaminyltransferases. The Ricin B domain, which is not involved (or required) for activity, appears to direct transferase activity to sites near to previous N-acetylgalactose substitution on the polypeptide (Fritz *et al.*, 2006). In this example, the DA is associated with 26 reviewed entries in UniProtKB/Swiss-Prot (during the training/learning step) with an F-score of 1.00, allowing propagation of this GO term to 2090 unreviewed entries in UniProtKB/TrEMBL, which would otherwise only receive the more general annotation associated with the two separate domains. Automatically annotating this GO term has particular value because InterPro does not provide any direct InterPro2GO mappings for this term. The reason is probably that InterPro does one-to-one mappings between entries and functional terms; however, here two different entries are required together for the function. This is a clear example of the value added by the DA approach.

**Table 3.** Two example cases where multiple domains are required for the defined protein function

GO id	GO term name	Associated Das	No. of training proteins	Association confidence (F-score)	No. of query annotated proteins
GO:0004653	polypeptide N-acetylgalactosaminyltransferase activity	1) GAP-IPR001173 -IPR000772 2) GAP-IPR001173 -GAP-IPR000772	26	1.00	5740

GO:0042813	Wnt-activated receptor activity	1) GAP-IPR020067-GAP-IPR0179812) IPR020067-GAP-IPR017981 3) IPR020067-IPR008993	25	0.91	1298

**Table 4.** Statistics and performance comparison between InterPro2GO and DAAC

	InterPro2GO	DAAC
Total no. of mappings	6382	25626
No. of unique entries	2927	8248
No. of unique GO terms	1411	778

No. of GO terms predicted by each system	1188	555
	(No. of shared terms: 223)	

No. of mapped GO term relations with the other system	760 in relation651 independent	625 in relation153 independent

% specificity of the mapped GO terms compared to other system	19%	75%
	(6% the same term)	

	**Performance comparison**	

F-score	0.675	0.874
Recall	0.615	0.843
Precision	0.909	0.919
FPR (fall-out)	1.98 × 10^−5^	4.57 × 10^−4^

The second example case: GO:0042813 (Wnt-activated receptor activity) is a molecular function category term that is associated with seven DAs (some are shown in Table 3), the DAs forming two distinct groups based on domain content. The interaction between Wnt proteins and frizzled receptor proteins forms a complex signaling pathway which plays an important role in embryogenesis, and is captured by this GO term. The protein domain responsible for Wnt binding is recognized by IPR0260067, and in most cases the proteins are membrane associated and contain a distinctive sequence of transmembrane domains recognized by the domain entry IPR017981. However, a significant number of proteins which have a role in modulating the signaling pathway are soluble, extracellular proteins belonging to the secreted frizzled-related protein (sFRP) family. The DA approach successfully groups all these proteins together, based on the common presence at the N terminus of the Wnt receptor domain IPR020067 followed in the sequence by either the transmembrane domain IPR017981 or IPR008993, a binding domain with beta-barrel topology, which is characteristically present in the sFRP family. The DAAC method therefore provides a more complete grouping of the proteins involved in the Wnt/frizzled signaling pathway than is achieved by using the domains separately and individually. In this example, the DA is associated with 25 reviewed entries in UniProtKB/Swiss-Prot (during the training/learning step) with a F1 score of 0.91, allowing propagation of this GO term to 1634 unreviewed entries in UniProtKB/TrEMBL .

### 3.5 Comparison with the state of the art

InterPro provides a semi-automatic annotation system called InterPro2GO where InterPro entries are one-to-one mapped to GO terms that defines the same property (Mitchell *et al.*, 2014). Then the sequences annotated with these InterPro entries receive the corresponding GO terms as predictions. InterPro2GO is also included in the UniProt GOA database. We compared the mappings of the DAAC method (between GO terms and DAs) with InterPro2GO to study the added value of using combinations of domains for defining specific functions over the single domain approach. This is not a comparison of the whole coverage of these systems on the protein universe where InterPro2GO is very advanced; but an observation of the specificity of protein function prediction that cannot be covered by the conventional function prediction approaches. After all, the DAAC method has been developed to work as a complementary method to the conventional systems currently used in UniProtKB database. This comparison is also reasonable because both methods utilize the same information as input (InterPro entries) and as a result, differences in the output will indicate the value of DAAC. Here, we only considered the mappings between domain type InterPro entries and GO terms for the comparison as DAAC only uses domains. It’s also assumed that the mappings between DAs/entries and GO terms do not contain errors. This can be justified by high performance in cross-validation for DAAC mappings and expert curation for InterPro2GO.

Table 4 shows the statistics of GO term mappings and the validation performance for both systems. As observed, InterPro2GO maps to more GO terms compared to DAAC. However, the number of mapped DAs is higher compared to those mapped to InterPro domain entries. This is due to mapping multiple very similar DAs to the same GO terms. In the DAG of GO, 760 mapped GO terms from InterPro2GO and 625 from DAAC have an ancestor-descendant relationship. We used these terms in order to compare the specificity of the predicted functions between the two systems. Considering all relations between the two groups of GO terms, we found that DAAC predicted GO terms are more specific in 75% of the cases. This indicates that multi-domain association approach is able to define more specific functions. In addition, 555 of the GO terms predicted by DAAC could not be predicted by InterPro2GO domain type entry associations (this number is 313 considering the whole InterPro2GO, including the GO term associations to the family type entries). These results indicate the significance of the value added to the protein function prediction in UniProt database by the DAAC method. The performance comparison between the two systems has been carried out considering the cross-validation of the systems for the 223 GO terms that both methods annotate, in order to obtain a fair comparison. As observed from Table 4, DAAC performed significantly better in terms of recall and overall F-score. InterPro2GO had a higher performance in terms of FPR, however, the precision of DAAC was slightly higher than that of InterPro2GO. An AUC comparison was not possible here since there is no scoring in InterPro2GO predictions and therefore it is not possible to draw ROC curves. The results indicate that the performance of DAAC is overall better than an already established and safely used automated GO annotation system i.e. InterPro2GO.

## 4 Conclusion and future work

In this article, we have proposed DAAC: a novel approach in the field of automatic functional annotation of protein sequences with the alignment and the classification of DAs. The proposed method is distinguished from conventional approaches in three main aspects: (i) the use of DAs as the basis of a similarity measure between proteins to propagate GO annotation; (ii) the employment of multi-label classification where each class represents a unique GO term, thus enabling the optimization of the parameters for each term independently and (iii) the use of InterPro as the domain resource in order to increase the coverage of domain annotation on the proteins (other novel points are discussed in the Supplementary information). DAAC enables the association of DAs with functional terms (each represented by a unique class) and the fast annotation of non-annotated proteins bearing the same or similar architectures. The employment of multi-label classification enables a protein (and its DA) to be a member of more than one class and thus have more than one functional annotation. Establishing independent classifiers for each GO term provides us with the ability to select different parameters for each class. With this approach we were able to optimize the class-specific DA similarity thresholds.

The performance of the method in the functional annotation of proteins was tested via cross-validation on the training dataset composed of UniProtKB/Swiss-Prot proteins, together with their experimentally validated GO annotation (F-score: 0.85). Next, the method was applied to nearly 55.3 million protein entries in UniProtKB/TrEMBL to obtain GO annotation for the whole database. This analysis resulted in 44 818 178 GO term predictions for 12 172 114 proteins, 2 812 016 of which were previously non-annotated. The results show that the proposed approach is effective and has the potential to identify functional relationships, especially between multi-domain proteins. Next, we plan to integrate the DAAC method into the UniProt automatic annotation production pipeline to enrich the automatic functional annotation of UniProtKB/TrEMBL. We also plan to extend the DAAC approach to the automatic annotation of EC numbers, UniProtKB keywords, UniProtKB comments, recommended protein names and subcellular locations.

## Supplementary Material

Supplementary Data
