# Supplementary material for: UniProt-DAAC: domain architecture alignment and classification, a new method for automatic functional annotation in UniProtKB
Source: Bioinformatics. 2016 Mar 7;32(15):2264–71. doi: 10.1093/bioinformatics/btw114 (PMC4965628; doi:10.1093/bioinformatics/btw114)
Supplement: Supplementary Data [file supp_32_15_2264__index.html]

UniProt-DAAC: Domain Architecture Alignment and Classification, a New Method for Automatic Functional Annotation in UniProtKB — UniProt-DAAC: domain architecture alignment and classification, a new method for automatic functional annotation in UniProtKB — UniProt-DAAC: domain architecture alignment and classification, a new method for automatic functional annotation in UniProtKB — Supplementary Data 

# UniProt-DAAC: domain architecture alignment and classification, a new method for automatic functional annotation in UniProtKB

## Supplementary Data

files

- Supplementary Data - docx file
