# Supplementary material for: UniProt-DAAC: domain architecture alignment and classification, a new method for automatic functional annotation in UniProtKB
Source: Bioinformatics. 2016 Mar 7;32(15):2264–71. doi: 10.1093/bioinformatics/btw114 (PMC4965628; doi:10.1093/bioinformatics/btw114)
Supplement: Supplementary Data [file supp_btw114_Supplementary_Data.docx]

**Supplementary Data**

## 1. Cross-validation and Performance Measures

A cross-validation experiment was carried out in order to study the performance of the proposed method. This is essentially a test-run using data with already known labels (the training data). UniProtKB/Swiss-Prot entries and their GO annotation with experimental evidence codes were used as the benchmarking set. However, in order to maintain an unbiased reference set, UniRef90 clusters have been used to avoid populating the dataset with highly similar sequences. One member from each UniRef90 cluster has been included in the benchmarking set. This procedure has prevented miscalculation of the performance by counting proteins with the exact same function (and attributes) more than once. The benchmark set proteins were tested in leave-one-out fashion where one entry is removed from the training set and the system is trained using the rest. The removed entry is then used as a query and the predictions are compared with its true labels. This procedure was repeated for all entries in the benchmark set and the performance was measured for each GO term class independently. The performance of the method in cross-validation was measured by recall, precision, F-score and area under the ROC curve (AUC). F-score is the harmonic mean of recall and precision. The ROC (Receiver Operating Characteristic) curve is the plot of False Positive Rate (FPR) vs. True Positives Rate (TPR), also known as recall. Recall, precision, FPR and F-score are defined as:

$R_{p}=\frac{{TP}_{p}}{{TP}_{p}+{FN}_{p}}$ (S1)

$P_{p}=\frac{{TP}_{p}}{{TP}_{p}+{FP}_{p}}$ (S2)

${FPR}_{p}=\frac{{FP}_{p}}{{FP}_{p}+{TN}_{p}}$ (S3)

$F_{p}=\frac{{2*TP}_{p}}{{2*TP}_{p}+{FN}_{p}+{FP}_{p}}$ (S4)

For a GO term class *p*: *R_p_* is recall; *P_p_* is precision; *FPR_p_* is Fase Positive Rate; *F_p_* is F-score; *TP_p_* (true positives) is the number of proteins that are correctly classified; *FP_p_* (false positives) is the number of proteins that are incorrectly classified into the class; *FN_p_* (false negatives) is the number of proteins that are incorrectly left out of the class; and *TN_p_* (true negatives) is the number of proteins that are correctly left out of the class.

**2. Performance Test Results of the Method**

Figure S1 displays the performance of the method in the cross-validation procedure, where each value on the horizontal axis represents a different GO term and the vertical axis corresponds to the performance measure for these terms in F-score. The black colored curve in Figure S1.A shows the overall performance of the method whereas the red curve displays the performance for the asserted annotations. Asserted annotations are the direct annotations of proteins (the most specific annotation from the same GO hierarchy). The rest are propagated annotations, which are generated by including all the terms higher up the hierarchy from the asserted annotation to the root of the GO Directed Acyclic Graph (DAG). As displayed in Figure S1.A, the performance of the method is better for asserted annotations than propagated annotations. This indicates that the method performs better on specific terms with a low number of associated proteins compared to generic terms. The reason is probably that the asserted annotations are specific and clear, and it is usually possible to find signatures in the protein sequence corresponding to the annotation. However, as we go towards the root, by automatically adding the parents of asserted annotations, the terms become more and more generic and finding clear signatures corresponding to these terms becomes difficult. Here, AUC calculation based on GO term classes was not possible since the annotations for each GO term are divided between asserted and propagated annotations.

In Figure S1.B the performance of the method is shown for different GO categories. As observed, the method performed best with molecular function terms (mean AUC: 0.908 ± 0.096), followed by cellular component terms (mean AUC: 0.846 ± 0.093), while the lowest performance was observed for biological process terms (mean AUC: 0.786 ± 0.099). The average performance with molecular function terms was 41% and 56% better than the average performance with cellular component and biological process terms respectively. These results are in accordance with previous studies where molecular function has been reported to be the easiest GO category to predict by automatic methods. This is again likely due to the closer and more direct relationship that exists between signatures found in the protein sequence and molecular function, compared to the relationship between the signatures and biological process that has a more complex nature. Biological process corresponds to a broader and generalized type of function compared to molecular function and it is highly probable that other types of contextual information are required to clearly define biological processes.

Fig S1. Cross-validation performance results in F-score: A) Comparison between asserted annotations and all annotations (including the propagated ones); B) Comparison between different GO categories.

A B

Figure S2 displays the cross-validation performance results in recall (for different GO term classes) to compare the effect of various settings (such as the addition of imaginary GAP domains in domain architectures and weighting the domains) on the performance. The figure is drawn in a similar fashion to Figure 2B and 2C in the main text, with the blue curve representing the overall method. Table S1 displays the mean performance for the same settings, via various measures. As observed from both Figure S2 and Table S1, the domain-weighting assumption did not have a significant effect; however, omitting the imaginary GAP domains caused an increase in recall. This result can be attributed to elevated similarity scores obtained without using the GAP domains (as the presence of GAP domains always decreases pairwise DA similarity), thus resulting in more predictions and less false negatives. Though, omitting GAP domains also increases the number of false positives and causes a reduced precision (as observed from Figure 2C in the main text). In the end, as also observed from Table S1, without the GAP domains, the balanced measure F-score decreases (from 0.85 to 0.63), along with the AUC (from 0.88 to 0.80).

Fig S2. Cross-validation results in Recall (to observe the effect of different settings on performance).

The reason behind obtaining a low number of GO terms within the high performance set (778 out of 13,826 terms) is the methodology we used in the performance calculation. We considered all positive and all negative samples for all GO term classes during the validation, instead of using a balanced positives and negatives set. This resulted in much larger negative sample sizes compared to the positives. The size of the negatives set is roughly proportional to the number of false positive hits one can obtain in this analysis. As a result, our precision results have fallen dramatically due to using unbalanced validation sets. However, we believe that the unbalanced set is the best representation of the unknown protein universe, as for a specific functional class the number of proteins that do not have this function is much greater than the number that do. In the end, the results we obtained using unbalanced sets are the best possible estimations of the performance of the method on the non-annotated/unknown set.

**Table S1.** Cross validation performance results considering different settings.

| **Performance (mean)** | **All assumptions (overall method)** | **No GAP domains** | **No domain weights** |
| --- | --- | --- | --- |
| F-score | 0.85 ± 0.09 | 0.63 ± 0.18 | 0.82 ± 0.13 |
| Recall | 0.84 ± 0.12 | 0.87 ± 0.12 | 0.83 ± 0.13 |
| Precision | 0.89 ± 0.14 | 0.54 ± 0.22 | 0.85 ± 0.19 |
| AUC | 0.88 ± 0.10 | 0.80 ± 0.10 | 0.87 ± 0.10 |

## 3. Other Novelties of DAAC

## One aspect of the novelty of the proposed approach is to use InterPro as the domain annotation source. Most of the previous studies about DAs have employed just the Pfam database (Finn, *et al.*, 2014). We preferred InterPro in order to increase the domain annotation coverage of the protein sequences since InterPro combines domain annotation from 11 different resources including Pfam.

Applying a domain weighting directly into the scoring matrix is proposed here for the first time. Previous approaches have applied domain weights following the alignment process (Terrapon, *et al.* 2014, Forslund, *et al.* 2011), where the weighting acts as a score normalization procedure. However, the optimal solution may not be obtained with these methods, especially for those proteins containing a high number of promiscuous domains, due to the equal treatment given to promiscuous domains compared to other domains during the alignment.

In our method the weights have already been applied before the dynamic programming stage; as a result, the optimal solution is always guaranteed in the alignment. In contrast to most previous DA alignment methods, the proposed method does not align the proteins with each other but aligns the architectures. All of the proteins with the same DA are grouped together and only one alignment is run for the whole group. This greatly reduces the total number of operations required to be run.

**Supplementary References**

Finn, R. D., *et al.* (2014). The Pfam protein families database. *Nucleic acids research, 42*, D222-D230.

Forslund, K., *et al.* (2011). Domain architecture conservation in orthologs. *BMC bioinformatics*, *12*(1), 326.

Terrapon, N., *et al.* (2014). Rapid similarity search of proteins using alignments of domain arrangements. *Bioinformatics, 30*(2), 274-281.
